# The prevalence of paramagnetic rim lesions in multiple sclerosis: A systematic review and meta-analysis

**DOI:** 10.1371/journal.pone.0256845

**Published:** 2021-09-08

**Authors:** Koy Chong Ng Kee Kwong, Daisy Mollison, Rozanna Meijboom, Elizabeth N. York, Agniete Kampaite, Michael J. Thrippleton, Siddharthan Chandran, Adam D. Waldman

**Affiliations:** Centre for Clinical Brain Sciences, University of Edinburgh, Edinburgh, United Kingdom; Henry Ford Health System, UNITED STATES

## Abstract

**Background:**

Recent findings from several studies have shown that paramagnetic rim lesions identified using susceptibility-based MRI could represent potential diagnostic and prognostic biomarkers in multiple sclerosis (MS). Here, we perform a systematic review and meta-analysis of the existing literature to assess their pooled prevalence at lesion-level and patient-level.

**Methods:**

Both database searching (PubMed and Embase) and handsearching were conducted to identify studies allowing the lesion-level and/or patient-level prevalence of rim lesions or chronic active lesions to be calculated. Pooled prevalence was estimated using the DerSimonian-Laird random-effects model. Subgroup analysis and meta-regression were performed to explore possible sources of heterogeneity. PROSPERO registration: CRD42020192282.

**Results:**

29 studies comprising 1230 patients were eligible for analysis. Meta-analysis estimated pooled prevalences of 9.8% (95% CI: 6.6–14.2) and 40.6% (95% CI: 26.2–56.8) for rim lesions at lesion-level and patient-level, respectively. Pooled lesion-level and patient-level prevalences for chronic active lesions were 12.0% (95% CI: 9.0–15.8) and 64.8% (95% CI: 54.3–74.0), respectively. Considerable heterogeneity was observed across studies (I^2^>75%). Subgroup analysis revealed a significant difference in patient-level prevalence between studies conducted at 3T and 7T (p = 0.0312). Meta-regression analyses also showed significant differences in lesion-level prevalence with respect to age (p = 0.0018, R^2^ = 0.20) and disease duration (p = 0.0018, R^2^ = 0.48). Other moderator analyses demonstrated no significant differences according to MRI sequence, gender and expanded disability status scale (EDSS).

**Conclusion:**

In this study, we show that paramagnetic rim lesions may be present in an important proportion of MS patients, notwithstanding significant variation in their assessment across studies. In view of their possible clinical relevance, we believe that clear guidelines should be introduced to standardise their assessment across research centres to in turn facilitate future analyses.

## Introduction

Susceptibility-based magnetic resonance imaging (MRI), which distinguishes tissues based on their magnetic susceptibility, is exquisitely sensitive to mineral content in the brain and has been applied to a variety of neurological conditions [1]. While phase images have historically been largely disregarded, they have recently gained renewed interest given their potential to inform about local susceptibility effects. Highlighting their significance, susceptibility-weighted imaging (SWI), a technique combining magnitude and phase information, offers excellent contrast between tissues of different magnetic susceptibilities, allowing the qualitative assessment of diamagnetic and paramagnetic features in the brain with high sensitivity [2]. Susceptibility-based imaging approaches also include quantitative susceptibility mapping (QSM), which allows absolute iron concentrations in tissues to be estimated [3].

Insights gained from combined pathological and radiological studies have resulted in an increasing interest in the potential application of susceptibility-based MRI in multiple sclerosis (MS) [4–6]. Classification systems for white matter lesions in MS have predominantly been derived from histological characterisation, broadly dividing them into ‘active’, ‘inactive’ and ‘remyelinated’ lesions based on their inflammatory cell distribution and demyelination profile [4, 7, 8]. However, brain tissue constituents such as myelin, iron and calcium have been found to generate susceptibility effects, raising the possibility that pathological changes in MS may be captured *in vivo* through SWI biomarkers [9, 10]. Detectable paramagnetic effects due to iron accumulation are associated with a range of neurodegenerative diseases including MS, and have generated particular attention in this regard [10, 11].

Pathological analysis of MS lesions that are surrounded by a rim of hypointense signal on susceptibility-based MRI has demonstrated corresponding iron deposition, with foci of activated myeloid cells around the lesion margins [9, 12, 13]. The presence of iron-enriched macrophages/microglia could potentially follow the uptake of iron released in response to insult to myelin and oligodendrocytes, although this remains to be established. These ‘rim’ lesions, which have been variously termed in the literature, have subsequently been suggested to reflect chronic inflammatory demyelination in MS patients [9, 12, 13].

Moreover, recent studies have shown that ‘chronic active lesions’, which are histologically characterised by ongoing demyelination at the edge of an inactive demyelinated core and are believed to predominate in progressive forms of MS [4, 14, 15], may be identified on MRI as non-gadolinium-enhancing rim lesions [13, 16]. MS patients with such ‘chronic active lesions’ have been found to experience earlier progression in disability and decreased brain volumes, indicating their potential prognostic significance [13]. Longitudinal imaging studies have also shown that rim lesions are more likely to expand over time compared with those without rims [12, 13], and slow expansion has been suggested as an alternative marker for chronic active demyelination, although this has been explored in only a few *in vivo* studies to date [17].

In addition to paramagnetic rims, susceptibility-based MRI has also revealed central veins within white matter lesions; both features appear to be highly specific to MS, and have therefore been proposed as possible diagnostic markers [18–20].

While the prevalence of the central vein sign has previously been systematically reviewed [21], an estimate of the pooled prevalence of paramagnetic rim lesions in MS patients has, to our knowledge, not been calculated to date. Here, we perform a systematic review and meta-analysis of the literature on rim lesions, in order to assess their prevalence both on a per-lesion basis and at a patient-level. Given the lack of consensus within the published literature, we conduct separate meta-analyses for rim lesions and chronic active lesions, which are defined as non-gadolinium enhancing rim lesions.

## Materials and methods

This systematic review was performed according to both the Preferred Reporting Items for Systematic Reviews and Meta-Analyses (PRISMA) statement [22] and the Meta-Analysis of Observational Studies in Epidemiology (MOOSE) group guidelines [23]. This study is registered with the International Prospective Register of Systematic Reviews (PROSPERO) as record number CRD42020192282.

### Search strategy

The following databases were searched on 26 June 2020 to identify studies investigating rim lesions in participants with clinically isolated syndrome (CIS) or MS: PubMed and Embase. Only studies for which the full-text article has been published in a peer-reviewed journal were eligible. Date of publication was restricted to the following period: 1 January 2000 to 1 June 2020. Eligibility was limited to studies published in the English language. Search terms included “multiple sclerosis”, “MS”, “magnetic resonance imaging”, “MRI”, “susceptibility”, “iron”, “rim” and “chronic active”, as well as related terms and abbreviations of these (S1 File). We further screened the reference lists of eligible studies and of relevant review articles for potential citations, after which all references were collated and transferred to Endnote X9 for removal of duplicates. An additional search was performed on 1 June 2021 to identify recently published studies, which were included in our sensitivity analyses.

### Study selection

Study selection was based on the following inclusion criteria: (a) patients with CIS or MS; (b) patients undergoing MRI including one of the following: SWI, QSM, FLAIR*, R2*, as well as any other T2*-dependent modalities deemed relevant; (c) outcomes of interest involving rim lesions or chronic active lesions (rim lesions were defined as T2-hyperintense lesions presenting with a partial or complete paramagnetic phase rim, while chronic active lesions were defined as non-gadolinium enhancing rim lesions); (d) provided data allowing for the calculation of patient-level prevalence and/or lesion-level prevalence of rim lesions or chronic active lesions. We excluded studies that met any of the following criteria: (a) review articles, qualitative studies, letters, editorials, opinions, and conference abstracts; (b) studies involving fewer than five participants; (c) studies based on post-mortem MRI; (d) studies involving cortical lesions; (e) animal studies. In the case of studies with overlapping patient populations, the earlier study was selected unless otherwise stated. Both prospective and retrospective studies were eligible.

The title and abstract of each paper were screened by one author (KCNKK). Full-text articles were retrieved for studies meeting the inclusion criteria and independently assessed by two authors (KCNKK and DM) for eligibility. Where a lack of concordance was observed, the final decision was taken following discussion with a third author (ADW).

### Data extraction

The primary outcomes for this study included the patient-level prevalence and the lesion-level prevalence of rim lesions and chronic active lesions (patient-level prevalence was defined as the proportion of patients with at least one rim lesion/chronic active lesion, while lesion-level prevalence was defined as the proportion of white matter lesions identified as rim lesions/chronic active lesions), data on which were extracted by one author (KCNKK) and reviewed by a second author (DM). A sample of cases were also discussed with a third author (ADW) to achieve consensus. Further data extracted include those pertaining to: (a) background characteristics (author/s, year of publication, country and institution, and study design); (b) imaging parameters (scanner model, scanner manufacturer, magnetic field strength and MRI sequence); (c) participant characteristics (number of participants, type of MS, gender, age, disease duration and expanded disability status scale (EDSS)). Additional details regarding data extraction are provided in S2 File.

### Quality assessment

We assessed individual studies by generating a list of adapted criteria based on the Joanna Briggs Institute Critical Appraisal Checklist for Analytical Cross-Sectional Studies [24] and the NIH Quality Assessment Tool for Observational Cohort and Cross-Sectional Studies [25]. Our ten criteria were as follows: (a) Were inclusion and exclusion criteria for the study sample clearly defined? (b) Were the study subjects and the setting adequately described? (c) Did the study recruitment involve a consecutive or random sample of patients? (d) Was the study design prospective in nature? (e) Was appropriate justification provided for the study sample size? (f) Were potential confounding factors identified by the study authors? (g) Was the lesion of interest (rim lesion or chronic active lesion) defined? (h) Was more than one investigator involved in identifying the lesion of interest? (i) Were intra-rater and inter-rater reliability assessed by the study authors? (j) Was the level of experience of investigators involved in identifying the lesion of interest (rim lesion or chronic active lesion) stated?

### Data synthesis

Data analysis was performed using the “meta” and “metafor” packages on the R statistical software, version 4.0.2. Prevalence values were calculated for individual studies and pooled using the DerSimonian-Laird random-effects model. Although the double arcsine transformation has previously been suggested to be more appropriate for stabilising the variance than the logit transformation [26], it has recently been shown to produce potentially erroneous results following back-transformation [27]. A logit transformation was therefore applied, with the pooled prevalence, along with 95% confidence interval (CI), being subsequently back-transformed to facilitate interpretation. Heterogeneity was assessed via Cochran’s Q and the I^2^ statistic, with values greater than 30%, 50% and 75% suggesting moderate, substantial and considerable heterogeneity, respectively [28]. Subgroup analyses were conducted according to field strength (3T vs 7T) and MRI sequence (Phase/SWI vs QSM vs T2*-weighted images) to explore possible sources of heterogeneity. Meta-regression analyses were also performed to assess the possible contribution of the following covariates: gender, age, disease duration and EDSS. Subgroup analysis and meta-regression were only conducted in our analysis of rim lesions due to the small number of studies involving chronic active lesions. Publication bias was assessed using funnel plots, with the statistical significance being evaluated using Egger’s test of bias. Finally, sensitivity analyses including studies that post-dated our literature search were performed to assess the robustness of our results.

## Results

### Search results

**Fig 1:**
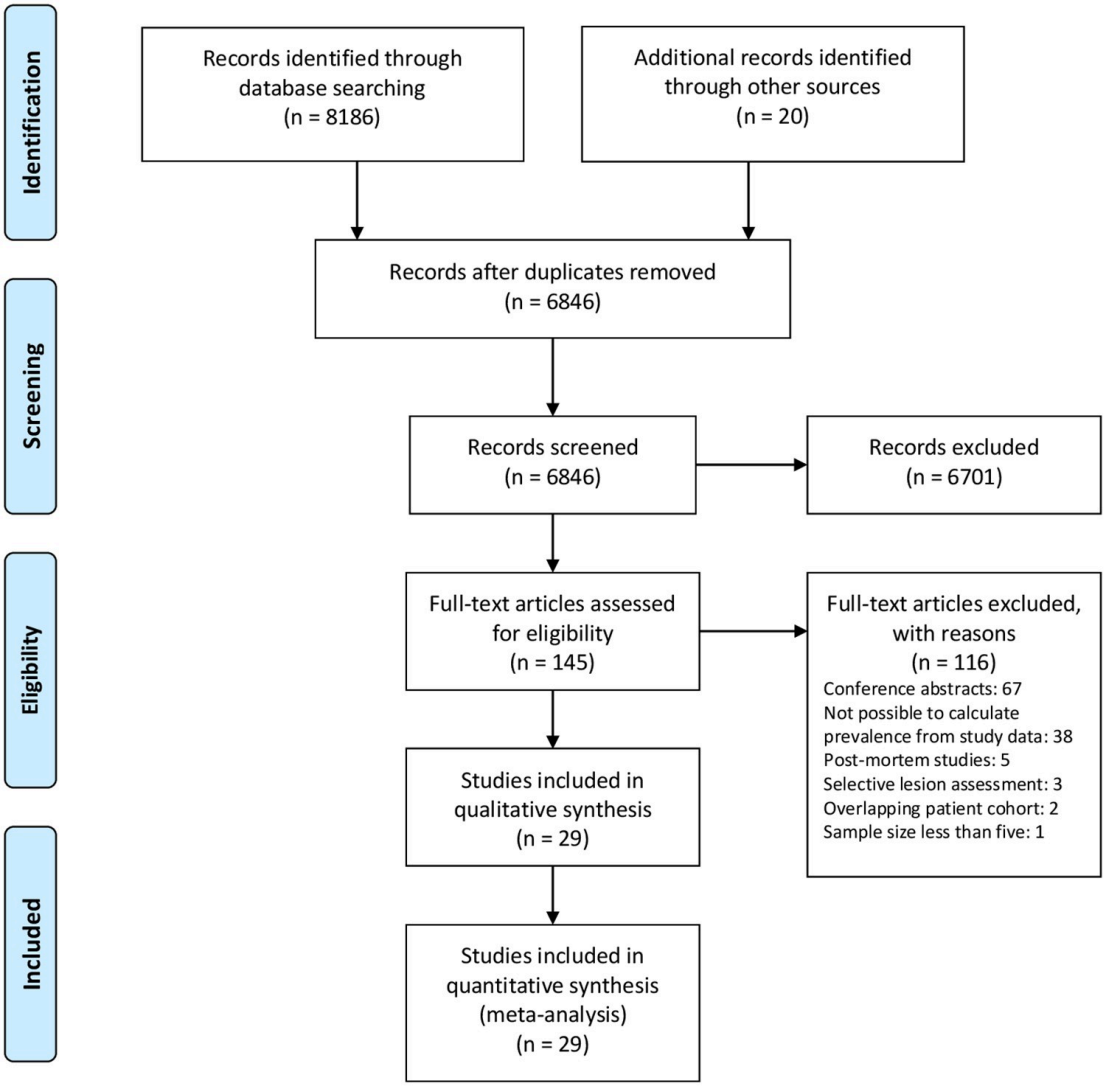
PRISMA flow diagram. PRISMA (Preferred Reporting Items for Systematic Reviews and Meta-Analyses) flow diagram outlining literature review and study selection.

8186 studies were identified by database searching, with 20 additional records obtained from handsearching (Fig 1). These reduced to 6846 studies following deduplication, and to 145 studies after screening title and abstract. After excluding 116 studies, such as those with overlapping patient populations and those in which insufficient data were provided to calculate prevalence, 29 studies remained eligible for analysis. We emailed 14 study investigators to request additional study data or to seek clarification about study methodology, and 7 kindly replied. Not captured by our search were 5 studies that post-dated our literature search [20, 29–32]. These were subsequently included in our sensitivity analyses.

### Study characteristics

Of the 29 included studies, 19 were conducted on 7T MRI systems, 8 involved 3T MRI systems, and the remaining 2 studies were performed at more than one field strength (Table 1). The paramagnetic rim sign was assessed using phase/SWI in 18 studies, QSM in 6 studies, and T2*-weighted images in 5 studies. MRI sequences such as FLAIR* and R2* were also used in 2 studies. 5 studies had a prospective study design while 8 studies were retrospective in nature. We were unable to ascertain the study design of the remaining studies. Finally, 8 studies focussed on chronic active lesions, whereas 21 studies did not restrict their analysis to non-gadolinium enhancing rim lesions.

**Table 1:** Study characteristics.

Author/s (Year of publication)	Institution (Country)	Study design	MRI sequence	Field strength	Scanner model (Manufacturer)	Lesion of interest
Hammond *et al*. (2008)	University of California San Francisco (USA)	NA	Phase/SWI	7T	Excite (GE)	Rim lesion
Haacke *et al*. (2009)	Wayne State University (USA)	NA	Phase/SWI	1.5T	Sonata (Siemens)	Rim lesion
				3T	Trio (Siemens)	
				4T	NA (Brucker/Siemens)	
Kollia *et al*. (2009)	University Hospital Essen (Germany)	NA	T2*-weighted images	7T	Magnetom (Siemens)	Rim lesion
Grabner *et al*. (2011)	Medical University of Vienna (Austria)	NA	Phase/SWI	7T	NA (Siemens)	Rim lesion
Suzuki *et al*. (2011)	Iwate Medical University (Japan)	Prospective	Phase/SWI	3T	Signa HDx (GE)	Rim lesion
Bian *et al*. (2012)	University of California San Francisco (USA)	NA	Phase/SWI	7T	NA (GE)	Rim lesion
Hagemeier *et al*. (2012)	University of Buffalo (USA)	NA	Phase/SWI	3T	Signa Excite HD 12.0 (GE)	Rim lesion
Sinnecker *et al*. (2012)	Charité-Universitaetsmedizin Berlin (Germany)	Retrospective	T2*-weighted images	7T	Magnetom (Siemens)	Rim lesion
Wuerfel *et al*. (2012)	Charité-Universitaetsmedizin Berlin (Germany)	Retrospective	T2*-weighted images	7T	Magnetom (Siemens)	Rim lesion
Yao *et al*. (2012)	National Institutes of Health (USA)	Retrospective	Phase/SWI	7T	NA (GE)	Chronic active lesion
Absinta *et al*. (2013)	National Institutes of Health (USA)	NA	Phase/SWI	7T	NA (Siemens)	Chronic active lesion
Mehta *et al*. (2013)	Ohio State University (USA)	NA	Phase/SWI	7T	Achieva (Philips)	Rim lesion
Kilsdonk *et al*. (2014)	VU University Medical Center & University Medical Center Utrecht (Netherlands)	Retrospective	FLAIR*	7T	Achieva (Philips)	Rim lesion
Kuchling *et al*. (2014)	Charité-Universitaetsmedizin Berlin (Germany)	NA	T2*-weighted images	7T	Magnetom (Siemens)	Rim lesion
Sati *et al*. (2014)	National Institutes of Health (USA)	NA	T2*-weighted images	3T	NA (Philips)	Rim lesion
Yao *et al*. (2015)	National Institutes of Health (USA)	NA	R2*	7T	Signa (GE)	Chronic active lesion
Absinta *et al*. (2016)	National Institutes of Health (USA)	Prospective	Phase/SWI	7T	NA (Siemens)	Chronic active lesion
Chawla *et al*. (2016)	New York University School of Medicine (USA) & Charité-Universitaetsmedizin Berlin (Germany)	NA	QSM	7T	Magnetom (Siemens)	Rim lesion
Cronin *et al*. (2016)	University of Nottingham (UK)	NA	Phase/SWI & QSM	7T	Achieva (Philips)	Rim lesion
Harrison *et al*. (2016)	Johns Hopkins University School of Medicine (USA)	Prospective	Phase/SWI & QSM	7T	Achieva (Philips)	Rim lesion
Sinnecker *et al*. (2016)	Charité-Universitaetsmedizin Berlin (Germany)	Retrospective	Phase/SWI	7T	Magnetom (Siemens)	Rim lesion
Dal Bianco *et al*. (2017)	Medical University of Vienna (Austria) & Vanderbilt University (USA)	Prospective	Phase/SWI	7T	Magnetom (Siemens)	Rim lesion
Chawla *et al*. (2018)	New York University School of Medicine (USA)	NA	QSM	7T	Magnetom (Siemens)	Rim lesion
Kaunzner *et al*. (2018)	Weill Cornell Medicine (USA)	Retrospective	QSM	3T	Signa HDxt (GE) or Magnetom Skyra (Siemens)	Chronic active lesion
Yao *et al*. (2018)	Weill Cornell Medicine (USA)	Retrospective	QSM	3T	Signa HDxt (GE)	Chronic active lesion
Absinta *et al*. (2019)	National Institutes of Health (USA)	Prospective	Phase/SWI	7T	Magnetom (Siemens)	Chronic active lesion
				3T	Skyra (Siemens)	
Eisele *et al*. (2019)	Universitaetsmedizin Mannheim (Germany)	Retrospective	Phase/SWI	3T	Magnetom Skyra (Siemens)	Chronic active lesion
Blindenbacher *et al*. (2020)	Charité-Universitaetsmedizin Berlin (Germany)	NA	Phase/SWI	3T	Trio TIM (Siemens)	Rim lesion
Clarke et al. (2020)	Vall d’Hebron University Hospital (Spain)	NA	Phase/SWI	3T	Magnetom Trio (Siemens)	Rim lesion

### Participant characteristics

The number of participants across studies ranged from 5 to 294, with study populations consisting of patients with different forms of MS, including relapsing-remitting MS (RRMS), secondary progressive MS (SPMS), primary progressive MS (PPMS), as well as CIS (Table 2). Most studies involved patients with RRMS, although patients with other forms of MS were also often included in the same study. The proportion of female participants ranged from 33.3% to 88.2%, while the mean age, mean disease duration and median EDSS ranged from 32 to 54.1 years, 1.5 to 17 years, and 1.5 to 3.8, respectively.

**Table 2:** Participant characteristics.

Author/s (Year of publication)	Number of participants	Type of MS	Gender (% female)	Age (years), Mean ± SD (Range)*	Disease duration (years), Mean ± SD (Range)*	EDSS, Median (Range)**	Patient-level prevalence	Lesion-level prevalence
Hammond *et al*. (2008)	19	RRMS	68.4%	42.32 ± 12.90	12.0 ± 7.6	2.1 ± 1.2^b^	-	7.7%
Haacke *et al*. (2009)	27	MS	77.8%	45 (21–71)	-	-	-	2.6%
Kollia *et al*. (2009)	12	RRMS	66.7%	32 (22–47)	5 (1–10)^a^	2.8 (1–3.5)	25%	-
Grabner *et al*. (2011)	10^#^	RRMS and SPMS	70%	41.6 (20–59)	11.8 (1–36)	3.3 (1–6.5)^b^	87.5%^#^	7.0%^#^
Suzuki et al. (2011)	11	RRMS	72.7%	32 (19–47)	4.6 (0.5–8.5)	-	-	33.8%
Bian *et al*. (2012)	5	RRMS	60%	51	17	3.1^b^	80%	6.7%
Hagemeier *et al*. (2012)	135	RRMS and SPMS	74.8%	46.7 ± 10.2	14.6 ± 9.8	3.5 ± 2.2^b^	22.2%	3.4%
Sinnecker *et al*. (2012)	18	RRMS	61.1%	41 ± 8 (27–53)	6.6 ± 5.8 (0.6–18.0)	1.5 (1.0–4.0)	-	23.3%
Wuerfel *et al*. (2012)	10	RRMS	60%	34 ± 6 (26–43)	4.0 ± 4.8 (0.5–14.3)	1.5 (0.0–4.0)	-	41.0%
Yao *et al*. (2012)	21	RRMS and SPMS	47.6%	45.2 ± 9.9 (28–60)	11.7 ± 9.5 (0.1–33)	2.1 (0–6)^§^	47.6%	7.3%
Absinta *et al*. (2013)	16	RRMS, SPMS and PPMS	87.5%	41.9 ± 10.8 (25–62)	7.8 (0.6–20)	2 (0–6)	75%	9.4%
Mehta *et al*. (2013)	16	RRMS and SPMS	-	46.8 ± 13.6 (28–66)	13.0 ± 11.1 (0.2–36)	3.6^§^	-	7.2% ^§^
Kilsdonk *et al*. (2014)	16	RRMS, SPMS and PPMS	62.5%	50.4 ± 3.9	10.4 ± 6.0 (2–22)	-	25%	2.3%
Kuchling *et al*. (2014)	18	RRMS and PPMS	33.3%	45.5 ± 5.9 (35–55)	6.4 ± 5.7 (0.2–16.8)	3.8 (1.5–8.0) ^§^	-	23.0%
Sati *et al*. (2014)	15	MS	33.3%	43 ± 13	-	-	6.7%	2.3%
Yao *et al*. (2015)	15	RRMS and SPMS	46.7%	44.8 ± 8.8 (28–57)	11.3 ± 10.1 (0.3–33)	1.5 (0–6.0)	66.7%	-
Absinta *et al*. (2016)	17	RRMS and SPMS	88.2%	40.9 ± 10.2 (29–62)	6.5 ± 6.4 (0.2–19)	1.5 (1–5.5)	88.2%	12.1%
Chawla *et al*. (2016)	21	RRMS and SPMS	71.4%	47.1 ± 10.3	11.5 ± 5.9 (4–25)	-	-	10.1%
Cronin *et al*. (2016)	39	CIS, RRMS, SPMS and PPMS	-	-	-	-	-	12.2% (Phase/SWI) 9.9% (QSM)
Harrison *et al*. (2016)	24	RRMS, SPMS and PPMS	50%	44.3 ± 10.0	11.2 ± 7.6	3.0 (1.5–6.5)	-	2.9% (Phase/SWI) 5.9% (QSM)
Sinnecker *et al*. (2016)	10	RRMS	50%	40 ± 7 (26–49)	6 ± 4 (0–12)	1.5 (0–2.5)	90%	32.3%
Dal Bianco *et al*. (2017)	8	RRMS and SPMS	62.5%	38.5 ± 15.1 (21–62)	11.6 ± 13.2 (3–37)	2 (0–6.5)	87.5%	15.3%
Chawla *et al*. (2018)	9	RRMS, SPMS and PPMS	66.7%	54.1 ± 13.3 (36.0 ± 70.3)	15.7 ± 12.4	2 (1–7)	33.3%	4.2%
Kaunzner *et al*. (2018)	30	RRMS and SPMS	60%	43.8 ± 14.3	13.1 ± 11.9	2.5 ± 2.3^b^	56.7%	10.6%
Yao *et al*. (2018)	46	CIS, RRMS and SPMS	69.6%	43.6 ± 10.7	8.7 ± 7.5	1.78 ± 1.84^b^	76.1%	20.8%
Absinta *et al*. (2019)	192	CIS, RRMS, SPMS and PPMS	68.8%	46.6 ± 12.8	13.0 ± 10.8	2 (0–8) ^§^	56.3%	-
Eisele *et al*. (2019)	294	RRMS and SPMS	76.9%	36 (18–69)	-	2.0 (0–7)	-	13.0%
Blindenbacher *et al*. (2020)	66	CIS and RRMS	60.6%	34 ± 8.6 (20–52)	1.5 ± 1.3 (0–4.3)	1.5 (0–4.5)	19.7%	4.6%
Clarke *et al*. (2020)	112	CIS	70.5%	35.4 ± 7.9 (19–49)	-	1.5 (0–4.5)	47.3%	19.9%

*Mean values provided unless otherwise indicated (Median values indicated by ^a^).

**Median values provided unless otherwise indicated (Mean values indicated by ^b^).

^§^Estimated from study data.

^#^Two subjects excluded from analysis.

Abbreviations: CIS = clinically isolated syndrome; EDSS = expanded disability status scale; MS = multiple sclerosis; PPMS = primary progressive multiple sclerosis; QSM = quantitative susceptibility mapping; RRMS = relapsing-remitting multiple sclerosis; SPMS = secondary progressive multiple sclerosis; SWI = susceptibility-weighted imaging.

### Quality assessment

Study participants were usually appropriately described, although not all studies clearly defined their inclusion and exclusion criteria. Studies also did not commonly recruit a consecutive or random sample of participants. More than half of included studies involved two or more raters in the visual assessment of rim lesions, but measures of intra-rater and inter-rater reliability were rarely reported. Many studies also provided the level of experience of study investigators. Details regarding quality assessment are provided in S1 Table.

### Lesion-level prevalence of rim lesions

**Fig 2:**
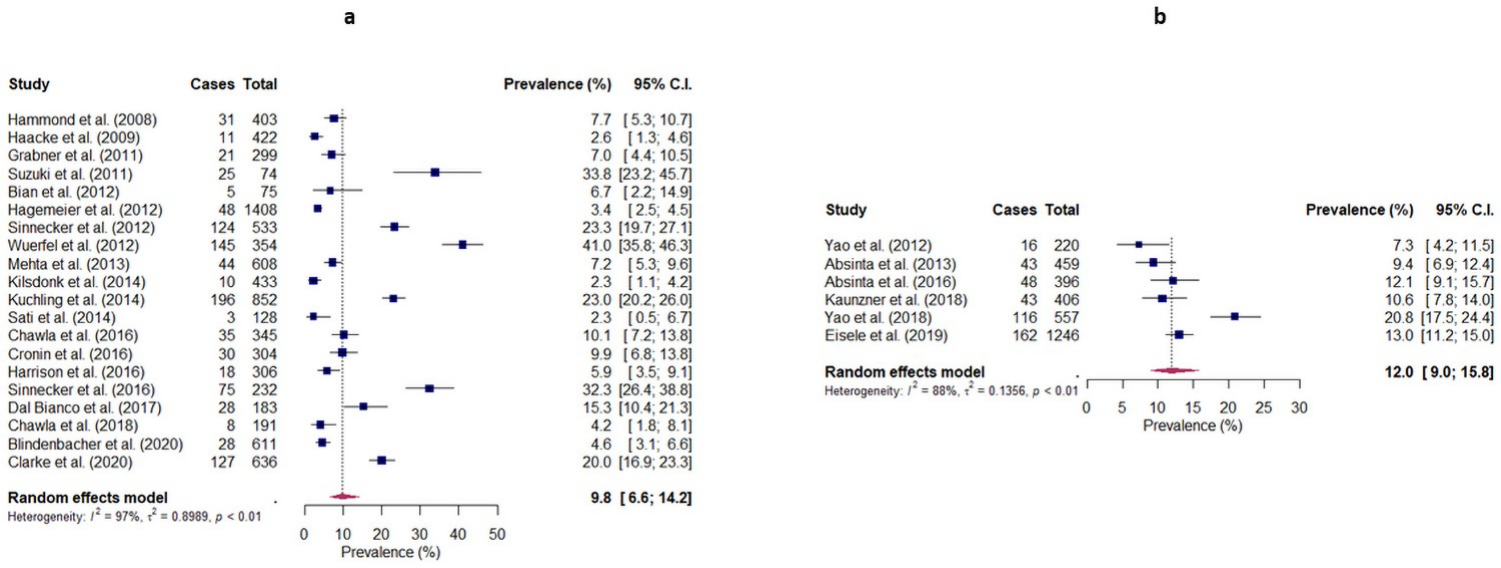
Random-effects forest plots showing the pooled lesion-level prevalence of (a) rim lesions and (b) chronic active lesions in patients with MS. The lesion-level prevalence observed by each study is represented by a square, with the 95% confidence interval being represented by a horizontal line. The pooled lesion-level prevalence is represented by a diamond, with the width corresponding to the 95% confidence interval. Cases = Number of identified rim lesions/chronic active lesions; Total = Total number of lesions.

The prevalence of rim lesions at the level of individual lesions was obtained for 20 studies, and ranged from 2.3% to 41.0% (Fig 2). Meta-analysis produced a pooled prevalence of 9.8% (95% CI: 6.6–14.2), with considerable heterogeneity being observed across studies (I^2^ = 97%, p<0.001). Subgroup analyses revealed no significant differences between studies conducted at different field strengths (3T: 8.5% (95% CI: 3.8–18.0), 7T: 11.1% (95% CI: 7.0–17.2), p = 0.561), and studies employing different MRI sequences (Phase/SWI: 9.6% (95% CI: 5.9–15.3), T2*-weighted images: 13.1% (95% CI: 6.4–24.9), QSM: 7.2% (95% CI: 3.1–15.7), p = 0.545). Meta-regression showed no statistically significant differences with respect to gender and EDSS (p = 0.578 and p = 0.143, respectively). However, statistically significant differences were observed regarding age (p = 0.0018, R^2^ = 0.20) and disease duration (p = 0.0018, R^2^ = 0.48), with the observed prevalence appearing to decrease with increasing age and disease duration. Age and disease duration were highly correlated across studies (Pearson’s r = 0.82, p<0.001). Egger’s test showed the presence of significant publication bias (p<0.001). Sensitivity analysis including three studies that post-dated our literature search produced a pooled prevalence of 10.2% (95% CI: 6.5–15.5).

### Lesion-level prevalence of chronic active lesions

The prevalence of chronic active lesions at the level of individual lesions was obtained for 6 studies, and ranged from 7.3% to 20.8% (Fig 2). Meta-analysis produced a pooled prevalence of 12.0% (95% CI: 9.0–15.8), with considerable heterogeneity being observed across studies (I^2^ = 88%, p<0.001). Egger’s test showed borderline publication bias (p = 0.0518).

### Patient-level prevalence of rim lesions

**Fig 3:**
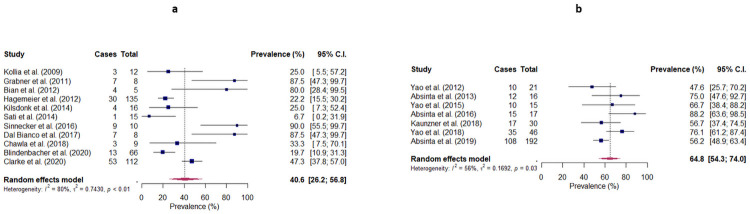
Random-effects forest plots showing the pooled patient-level prevalence of (a) rim lesions and (b) chronic active lesions in patients with MS. The patient-level prevalence observed by each study is represented by a square, with the 95% confidence interval being represented by a horizontal line. The pooled patient-level prevalence is represented by a diamond, with the width corresponding to the 95% confidence interval. Cases = Number of patients with at least one rim lesion/chronic active lesion; Total = Total number of patients.

The prevalence of rim lesions at patient-level was obtained for 11 studies, and ranged from 6.7% to 90.0% (Fig 3). Meta-analysis produced a pooled prevalence of 40.6% (95% CI: 26.2–56.8), with considerable heterogeneity being observed across studies (I^2^ = 80%, p<0.001). Subgroup analyses revealed a significant difference between studies conducted at different field strengths (3T: 24.4% (95% CI: 11.2–45.4), 7T: 57.6% (95% CI: 35.5–77.1), p = 0.0312), but no significant difference between studies employing different MRI sequences (Phase/SWI: 53.2% (95% CI: 32.8–72.6), T2*-weighted images: 18.7% (95% CI: 5.7–46.5), QSM: 33.3% (95% CI: 5.0–82.7), p = 0.132). Meta-regression showed no statistically significant differences with respect to gender, age, disease duration and EDSS (p = 0.845, p = 0.975, p = 0.568 and p = 0.484, respectively). Egger’s test showed the presence of significant publication bias (p = 0.0295). Sensitivity analysis including three studies that post-dated our literature search produced a pooled prevalence of 46.5% (95% CI: 33.1–60.5).

### Patient-level prevalence of chronic active lesions

The prevalence of chronic active lesions at patient-level was obtained for 7 studies, and ranged from 47.6% to 88.2% (Fig 3). Meta-analysis produced a pooled prevalence of 64.8% (95% CI: 54.3–74.0), with substantial heterogeneity being observed across studies (I^2^ = 56%, p = 0.035). Egger’s test showed no evidence of publication bias (p = 0.109). Sensitivity analysis including one study that post-dated our literature search produced a pooled prevalence of 61.3% (95% CI: 53.1–69.0).

## Discussion

In this study, we estimated pooled prevalences of 9.8% and 40.6% for rim lesions at lesion-level and patient-level, respectively. The pooled lesion-level and patient-level prevalences for chronic active lesions were 12.0% and 64.8%, respectively. Studies conducted using 7T MRI systems observed a significantly higher prevalence of rim lesions at patient-level than those performed at 3T MRI. Notably, the prevalence of rim lesions on a per-lesion basis was significantly lower in studies involving older patients or patients with longer disease durations. However, factors such as MRI sequence, gender and EDSS, did not appear to influence the prevalence observed across studies.

Our findings, which indicate that paramagnetic rim lesions are present in about half of MS patients and a small, though appreciable, proportion of individual MS lesions (about one in ten), are consistent with recent findings showing that rim lesions may be less prevalent than central veins both at patient-level and lesion-level [18, 20], the latter estimated at more than 70% of MS lesions [21]. Although this highlights obvious challenges with using rim lesions as a lone diagnostic biomarker, they nevertheless retain important clinical relevance given their specificity to MS and association with disease severity. It has thus been proposed that rim lesions could be coupled to central veins as a combined biomarker to improve both the diagnosis and prognosis of MS [33].

While the patient-level prevalence of rim lesions was found to be independent of age and disease duration, it remains unclear from our systematic review why studies involving patients with longer disease duration observe fewer paramagnetic rims on a per-lesion basis. The lack of correlation between disease duration and total lesion load (Pearson’s r = 0.012, p = 0.97) indicates that this is unlikely to be driven by an increase in total lesion count. Whether this effect could reflect more widespread inflammatory activity during early stages of the disease remains to be established, although it should be noted that the majority of the studies comprised patient cohorts with established or longstanding disease, and published data regarding the prevalence of rim lesions at the point of clinical presentation are limited. The possibility that this finding is due to the relatively low disability scores observed across cohorts included in our analysis also cannot be ruled out, and that these may not be fully representative of progressive phenotypes associated with higher disability levels. Future longitudinal studies are required to establish whether rim lesions may be a truly transient phenomenon in early disease, or if such associations result from other features of the subject groups.

Susceptibility-based MRI is a relatively recent addition to the MS imaging research toolbox, and there is currently considerable variation regarding visual assessment of the paramagnetic rim sign. We noted different criteria being applied across studies when evaluating rim lesions; some required the presence of a paramagnetic rim on at least three contiguous slices, and others restricted inclusion criteria to those with complete rims. Because posterior fossa brain structures are particularly prone to SWI artifacts, many studies included only supratentorial lesions in their analysis. It has previously been observed that assessment of rim lesions may itself be highly subjective, and there are no current widely accepted guidelines on what constitutes a paramagnetic rim [34].

A number of different imaging techniques have been employed to study rim lesions, and there is additional substantial variation in specific sequences and parameters used for SWI, QSM and T2*-weighted acquisition, processing and analysis. Evidence from individual comparative studies suggests that different MRI sequences may not be equally sensitive and specific to paramagnetic rims; for example, fewer rims are observed with QSM compared with SWI [35, 36]. Although our subgroup analyses showed significant effects of MRI field strength at patient-level, we did not find differential sensitivity across the type of susceptibility-based imaging technique used. We suspect this may be due to factors such as the varying criteria used to define rim lesions.

While similar prevalence was observed at 3T and 7T on a per-lesion basis, a finding consistent with the literature [34], the lower sensitivity of 3T MRI observed at patient-level deserves further investigation, given its implications for clinical practice. Although the patient-level prevalence of rim lesions also appeared greater using phase/SWI (53.2%) than on T2*-weighted images (18.7%) and QSM (33.3%), this did not reach statistical significance, possibly as a result of inadequate statistical power. Lastly, the total lesion load recorded across studies was based on different MRI sequences, which, coupled with variation in the way that lesion load was defined, could also have contributed to differences in the observed prevalence of rim lesions on a per-lesion basis. Notwithstanding these limitations, we believe that these results provide reasonable estimates of how common rim lesions and the subgroup that represent chronic active lesions are in MS patients, with only minor deviations observed upon inclusion of newly published studies in our sensitivity analyses.

Given that a major proportion of studies included patients with more than one subtype of MS, we were unable to assess the difference in prevalence between patients with RRMS and those with progressive forms of disease. Although individual studies have attempted to investigate possible differences, additional evidence is required to draw definite conclusions [7, 37]. We were also unable to account for the effect of disease-modifying therapy as a possible confounding factor in our meta-analysis.

We recognise a number of additional limitations to the data available for our review. Only a relatively small proportion of included studies were conducted prospectively, others being potentially vulnerable to various sources of bias due to their retrospective design. We further noted that primary study aims varied considerably, which could contribute to differences in the observed prevalence of rim lesions. Funnel plots and Egger’s tests were also suggestive of significant publication bias. Given that the prevalence of chronic active lesions was only assessed by a small number of studies, we were unable to conduct appropriate moderator analyses. Finally, it should be emphasised that subgroup and meta-regression analyses are by their nature observational, and results must therefore be interpreted with caution.

## Conclusion

Our systematic review suggests that paramagnetic rim lesions are present in a substantial proportion of MS patients, notwithstanding considerable heterogeneity in their assessment across studies. While evidence indicates that they could potentially play a prognostic role in MS, prevalence on a patient and lesion level may limit sensitivity for initial diagnosis. We believe there is a need to establish clear criteria for evaluation of rim lesions, which will improve interpretation of data acquired across different research centres. This, combined with harmonised acquisition protocols, will facilitate larger scale prospective multicentre studies across the disease course, which will allow validation and potential translation of this imaging marker into future clinical practice.

## Supporting information

S1 Fig Example rim lesion. (TIF)

S1 Table Quality assessment. (DOCX)

S1 File Search strategy. (DOCX)

S2 File Data extraction. (DOCX)

S3 File Subgroup analysis and meta-regression. (DOCX)

S4 File Funnel plots. (DOCX)

S5 File Sensitivity analysis. (DOCX)

S6 File PRISMA checklist. (DOCX)

## Abbreviations

CIS: clinically isolated syndrome
DMT: disease-modifying therapy
EDSS: expanded disability status scale
MOOSE: Meta-Analysis of Observational Studies in Epidemiology
MRI: magnetic resonance imaging
PPMS: primary progressive multiple sclerosis
PRISMA: Preferred Reporting Items for Systematic Reviews and Meta-Analyses
PROSPERO: International Prospective Register of Systematic Reviews
QSM: quantitative susceptibility mapping
RRMS: relapsing-remitting multiple sclerosis
SPMS: secondary progressive multiple sclerosis
SWI: susceptibility-weighted imaging
