# Supplementary figures and images for: The prevalence of paramagnetic rim lesions in multiple sclerosis: A systematic review and meta-analysis
Source: PLoS One. 2021 Sep 8;16(9):e0256845. doi: 10.1371/journal.pone.0256845 (PMC8425533; doi:10.1371/journal.pone.0256845)

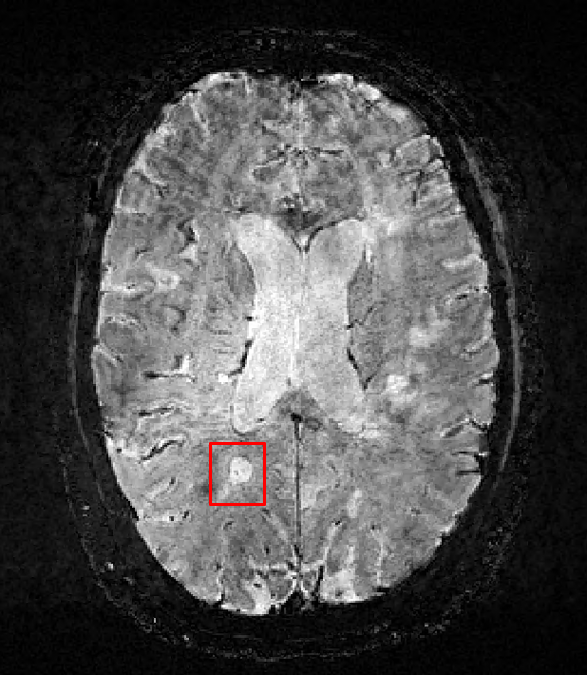

Supplement: S1 Fig — (TIF) [file pone.0256845.s001.tif]
