# Supplementary material for: The prevalence of paramagnetic rim lesions in multiple sclerosis: A systematic review and meta-analysis
Source: PLoS One. 2021 Sep 8;16(9):e0256845. doi: 10.1371/journal.pone.0256845 (PMC8425533; doi:10.1371/journal.pone.0256845)
Supplement: S1 Table — (DOCX) [file pone.0256845.s002.docx]

**S1 Table. Quality assessment**

| **Question** | **Were inclusion and exclusion criteria for the study sample clearly defined?** | **Were the study subjects and the setting adequately described?** | **Did the study recruitment involve a consecutive or random sample of patients?** | **Was the study design prospective in nature?** | **Was appropriate justification provided for the study sample size?** | **Were potential confounding factors identified by the study authors?** | **Was the lesion of interest (rim lesion or chronic active lesion) defined?** | **Was more than one investigator involved in identifying the lesion of interest?** | **Were intra-rater and/or inter-rater reliability assessed by the study authors?** | **Was the level of experience of investigators involved in identifying the lesion of interest stated?** |
| --- | --- | --- | --- | --- | --- | --- | --- | --- | --- | --- |
| Hammond *et al.* (2008) | X | ✓ | X | X | X | ✓ | ✓ | X | X | X |
| Haacke *et al.* (2009) | X | ✓ | X | X | X | X | ✓ | ✓ | X | ✓ |
| Kollia *et al.* (2009) | X | ✓ | ✓ | X | X | ✓ | ✓ | X | X | X |
| Grabner *et al.* (2011) | ✓ | ✓ | X | X | X | ✓ | ✓ | X | X | ✓ |
| Suzuki *et al.* (2011) | ✓ | ✓ | ✓ | ✓ | X | ✓ | ✓ | ✓ | ✓ | ✓ |
| Bian *et al.* (2012) | X | ✓ | X | X | X | ✓ | ✓ | X | X | X |
| Hagemeier *et al.* (2012) | X | ✓ | ✓ | X | X | ✓ | ✓ | X | X | X |
| Sinnecker *et al.* (2012) | X | ✓ | X | X | X | ✓ | ✓ | ✓ | X | ✓ |
| Wuerfel *et al.* (2012) | X | ✓ | ✓ | X | X | ✓ | ✓ | X | X | X |
| Yao *et al.* (2012) | ✓ | ✓ | X | X | X | ✓ | ✓ | ✓ | X | ✓ |
| Absinta *et al.* (2013) | X | ✓ | ✓ | X | X | ✓ | ✓ | ✓ | X | ✓ |
| Mehta *et al.* (2013) | X | ✓ | X | X | X | ✓ | ✓ | X | X | X |
| Kilsdonk *et al.* (2014) | X | ✓ | X | X | X | ✓ | ✓ | ✓ | X | ✓ |
| Kuchling *et al.* (2014) | X | ✓ | X | X | X | ✓ | ✓ | ✓ | ✓ | ✓ |
| Sati *et al.* (2014) | X | ✓ | X | X | X | X | ✓ | X | X | X |
| Yao *et al.* (2015) | ✓ | ✓ | ✓ | X | X | ✓ | ✓ | ✓ | X | X |
| Absinta *et al.* (2016) | X | ✓ | X | ✓ | X | ✓ | ✓ | ✓ | X | ✓ |
| Chawla *et al.* (2016) | X | ✓ | X | X | X | ✓ | ✓ | X | X | X |
| Cronin *et al.* (2016) | X | X | X | X | X | X | ✓ | X | X | X |
| Harrison *et al.* (2016) | X | ✓ | X | ✓ | X | ✓ | ✓ | X | X | X |
| Sinnecker *et al.* (2016) | X | ✓ | X | X | X | ✓ | ✓ | ✓ | ✓ | ✓ |
| Dal Bianco *et al.* (2017) | ✓ | ✓ | ✓ | ✓ | X | ✓ | ✓ | ✓ | X | ✓ |
| Chawla *et al.* (2018) | X | ✓ | X | X | X | ✓ | ✓ | ✓ | X | ✓ |
| Kaunzner *et al.* (2018) | X | ✓ | X | X | X | ✓ | ✓ | ✓ | X | ✓ |
| Yao *et al.* (2018) | ✓ | ✓ | X | X | X | ✓ | ✓ | ✓ | X | ✓ |
| Absinta *et al.* (2019) | ✓ | ✓ | ✓ | ✓ | X | ✓ | ✓ | ✓ | X | X |
| Eisele *et al.* (2019) | ✓ | ✓ | X | X | X | ✓ | ✓ | ✓ | X | ✓ |
| Blindenbacher *et al.* (2020) | ✓ | ✓ | X | X | X | ✓ | ✓ | ✓ | X | ✓ |
| Clarke *et al.* (2020) | ✓ | ✓ | ✓ | X | X | ✓ | ✓ | ✓ | ✓ | ✓ |
