# Supplementary material for: The prevalence of paramagnetic rim lesions in multiple sclerosis: A systematic review and meta-analysis
Source: PLoS One. 2021 Sep 8;16(9):e0256845. doi: 10.1371/journal.pone.0256845 (PMC8425533; doi:10.1371/journal.pone.0256845)
Supplement: S1 File — (DOCX) [file pone.0256845.s003.docx]

**S1 File. Search strategy**

Date: 26 June 2020

PubMed

Multiple sclerosis OR MS OR RRMS OR PPMS OR SPMS OR clinically isolated syndrome OR CIS

Susceptibility OR susceptibility weighted imaging OR SWI OR quantitative susceptibility mapping OR QSM OR phase OR gradient echo OR GE

Rim OR ring OR shell OR annular OR chronic inflammat* OR chronic active OR smoulder* OR smolder* OR enlarg* OR expan* OR erod* OR eros* OR evol* OR iron OR paramagnetic

1 January 2000 to 1 June 2020

| **#** | **Searches** | **Results** |
| --- | --- | --- |
| 1 | Multiple sclerosis[Title/Abstract] OR MS[Title/Abstract] OR RRMS[Title/Abstract] OR PPMS[Title/Abstract] OR SPMS[Title/Abstract] OR clinically isolated syndrome[Title/Abstract] OR CIS[Title/Abstract] | 501,374 |
| 2 | Susceptibility[Title/Abstract] OR susceptibility weighted imaging[Title/Abstract] OR SWI[Title/Abstract] OR quantitative susceptibility mapping[Title/Abstract] OR QSM[Title/Abstract] OR phase[Title/Abstract] OR gradient echo[Title/Abstract] OR GE[Title/Abstract] | 1,266,047 |
| 3 | Rim[Title/Abstract] OR ring[Title/Abstract] OR shell[Title/Abstract] OR annular[Title/Abstract] OR chronic inflammat*[Title/Abstract] OR chronic active[Title/Abstract] OR smoulder*[Title/Abstract] OR smolder*[Title/Abstract] OR enlarg*[Title/Abstract] OR expan*[Title/Abstract] OR erod*[Title/Abstract] OR eros*[Title/Abstract] OR evol*[Title/Abstract] OR iron[Title/Abstract] OR paramagnetic[Title/Abstract] | 1,661,925 |
| 4 | 1 AND 2 AND 3 | 4,562 |
| 5 | 4 AND ("2000/01/01"[Date - Publication] : "2020/06/01"[Date - Publication]) | 4,082 |
| 6 | 5 AND (English[Language]) | 4,013 |

Embase

Multiple sclerosis OR MS OR RRMS OR PPMS OR SPMS OR clinically isolated syndrome OR CIS

Susceptibility OR SWI OR QSM OR phase OR gradient echo OR GE

Rim OR ring OR shell OR annular OR chronic inflammat* OR chronic active OR smoldering OR smouldering OR enlarging OR expanding OR eroding OR evolving OR iron OR paramagnetic

| **#** | **Searches** | **Results** |
| --- | --- | --- |
| 1 | (Multiple sclerosis or MS or RRMS or PPMS or SPMS or clinically isolated syndrome or CIS).mp. | 673,791 |
| 2 | limit 1 to (english language and yr="2000 - 2020") | 553,581 |
| 3 | (Susceptibility or SWI or QSM or phase or gradient echo or GE).mp. | 1,835,697 |
| 4 | limit 3 to (english language and yr="2000 - 2020") | 1,447,479 |
| 5 | (Rim or ring or shell or annular or chronic inflammat* or chronic active or smoldering or smouldering or enlarging or expanding or eroding or evolving or iron or paramagnetic).mp. | 896,878 |
| 6 | limit 5 to (english language and yr="2000 - 2020") | 712,018 |
| 7 | 2 AND 4 AND 6 | 4,173 |
