# Supplementary material for: The prevalence of paramagnetic rim lesions in multiple sclerosis: A systematic review and meta-analysis
Source: PLoS One. 2021 Sep 8;16(9):e0256845. doi: 10.1371/journal.pone.0256845 (PMC8425533; doi:10.1371/journal.pone.0256845)
Supplement: S2 File — (DOCX) [file pone.0256845.s004.docx]

**S2 File. Data extraction**

In studies where gadolinium enhancement was performed, the lesion of interest was classed as ‘rim lesion’ or ‘chronic active lesion’ according to the primary aim of the study. To maintain consistency across studies, the lesion-level prevalence of chronic active lesions was calculated as the number of chronic active lesions divided by the total number of chronic or non-gadolinium enhancing lesions. Due to the wide-ranging imaging protocols employed across studies, we decided to categorise MRI sequences as ‘Phase/SWI’, ‘QSM’, ‘T2*-weighted images’, ‘FLAIR*’ and ‘R2*’. In cases where both phase/SWI and QSM values were reported, both have been included in Table 1, although QSM values were used in our quantitative analysis. Studies for which the study design (prospective or retrospective) could not be ascertained were labelled as ‘NA’ unless stated otherwise. Finally, where studies performed a longitudinal assessment of rim lesions, baseline values were used.
