# Supplementary material for: The prevalence of paramagnetic rim lesions in multiple sclerosis: A systematic review and meta-analysis
Source: PLoS One. 2021 Sep 8;16(9):e0256845. doi: 10.1371/journal.pone.0256845 (PMC8425533; doi:10.1371/journal.pone.0256845)
Supplement: S3 File — (DOCX) [file pone.0256845.s005.docx]

**S3 File. Subgroup analysis and meta-regression**


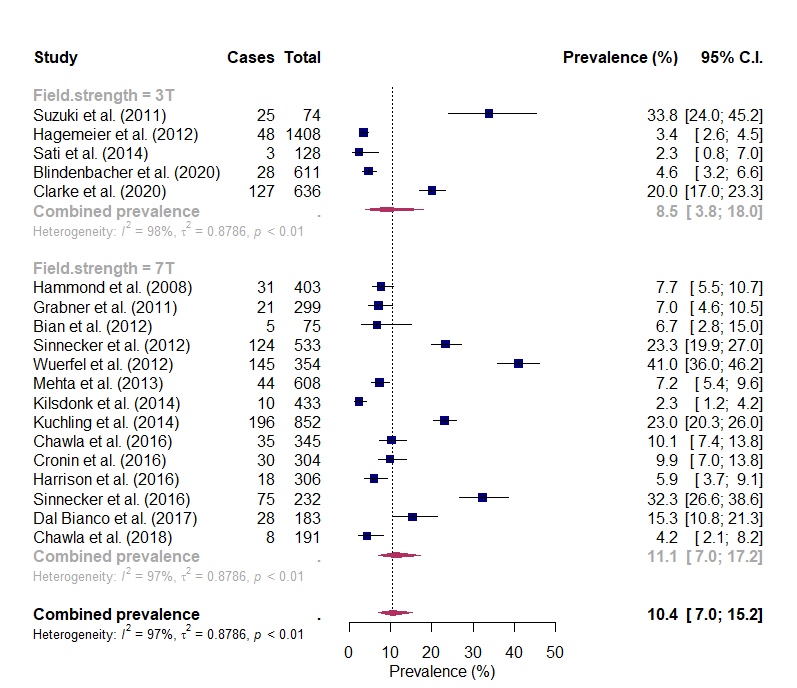


**Fig S3a.** Subgroup analysis of the lesion-level prevalence of rim lesions according to field strength (3T vs 7T).


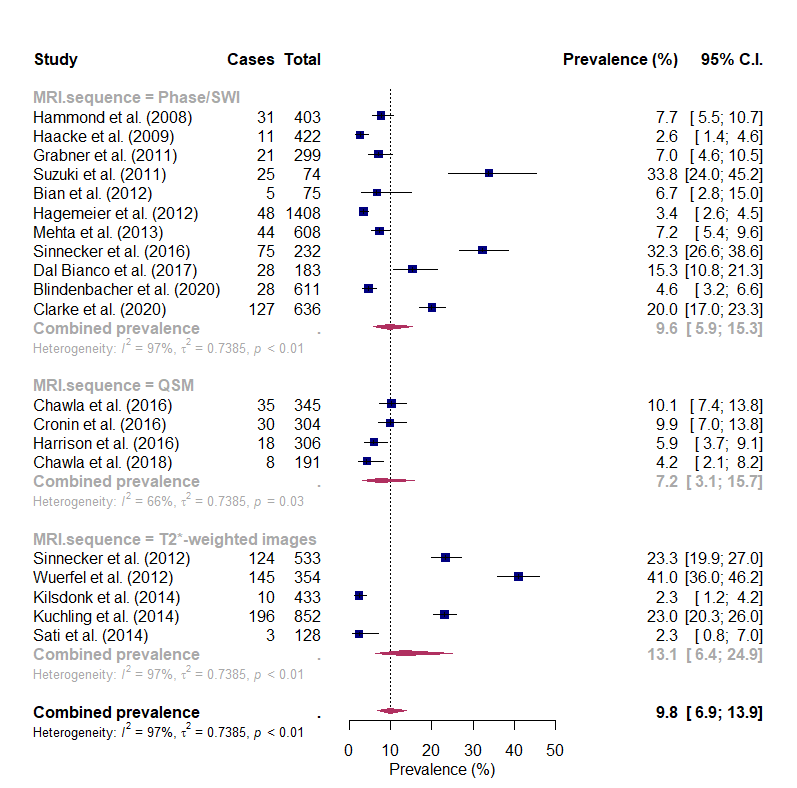


**Fig S3b.** Subgroup analysis of the lesion-level prevalence of rim lesions according to MRI sequence (Phase/SWI vs QSM vs T2*-weighted images).


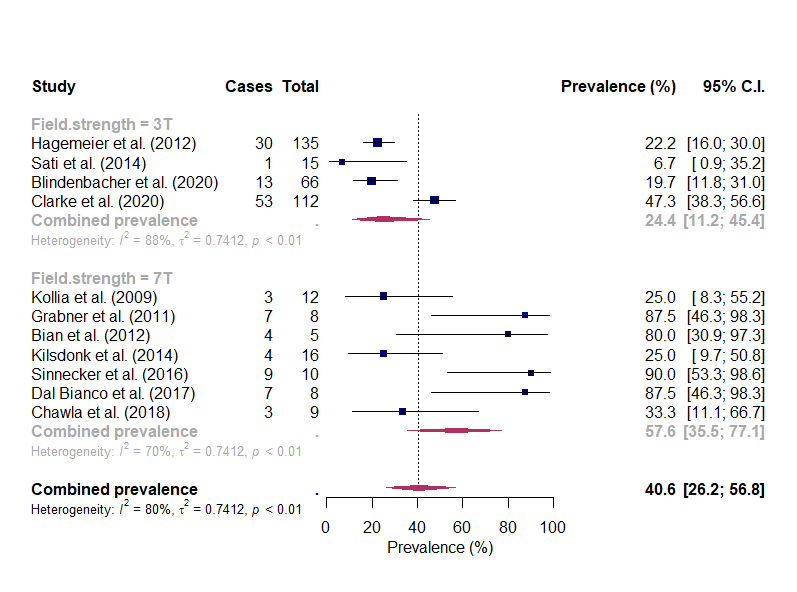


**Fig S3c.** Subgroup analysis of the patient-level prevalence of rim lesions according to field strength (3T vs 7T).


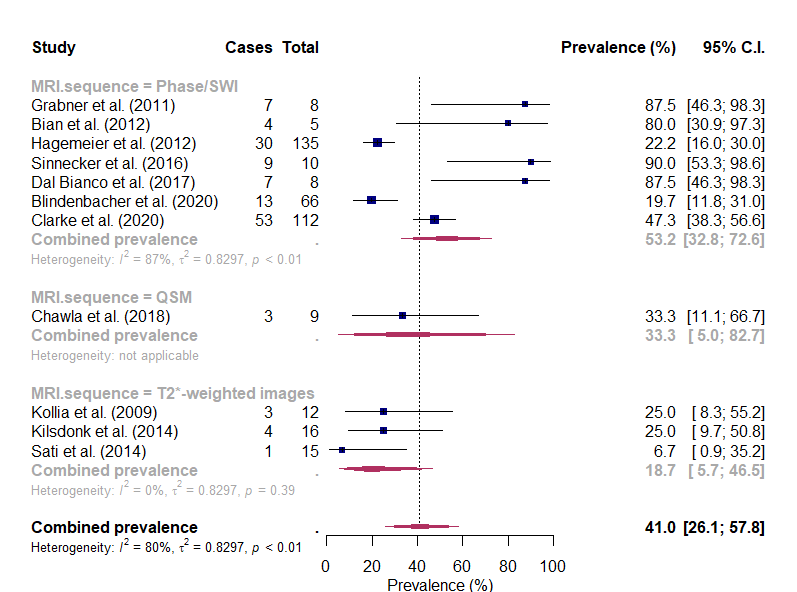


**Fig S3d.** Subgroup analysis of the patient-level prevalence of rim lesions according to MRI sequence (Phase/SWI vs QSM vs T2*-weighted images).


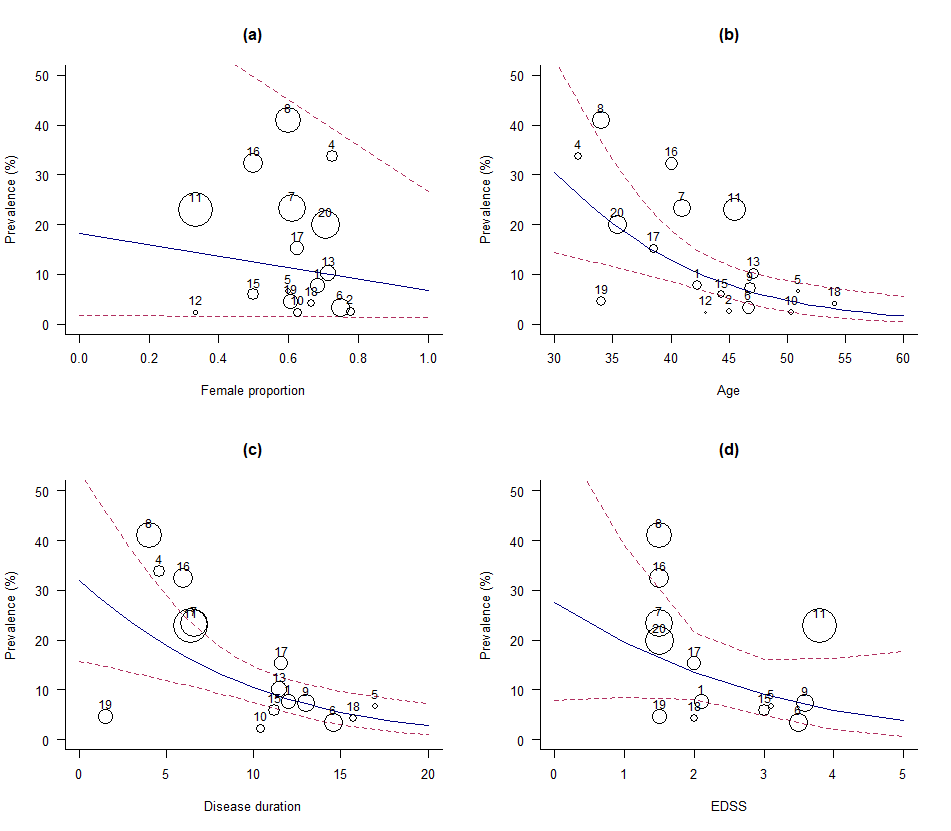


**Fig S3e.** Meta-regression analyses of the lesion-level prevalence of rim lesions according to (a) gender (b) age (c) disease duration and (d) EDSS.


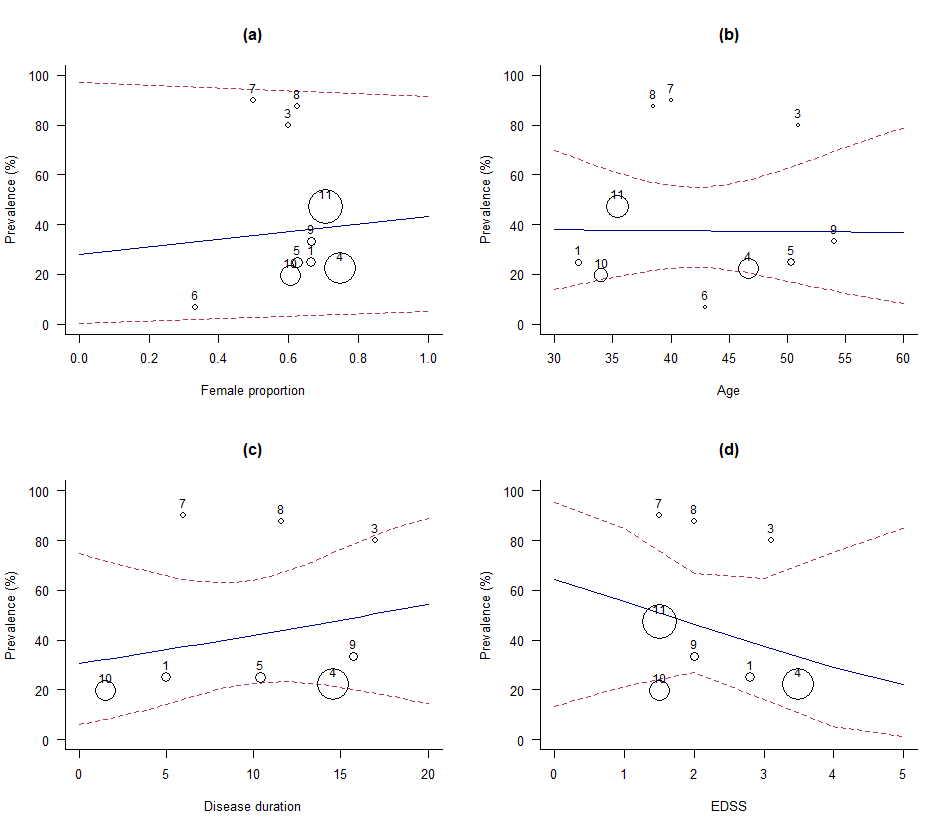


**Fig S3f.** Meta-regression analyses of the patient-level prevalence of rim lesions according to (a) gender (b) age (c) disease duration and (d) EDSS.
