# Supplementary material for: The prevalence of paramagnetic rim lesions in multiple sclerosis: A systematic review and meta-analysis
Source: PLoS One. 2021 Sep 8;16(9):e0256845. doi: 10.1371/journal.pone.0256845 (PMC8425533; doi:10.1371/journal.pone.0256845)
Supplement: S4 File — (DOCX) [file pone.0256845.s006.docx]

**S4 File. Funnel plots.**


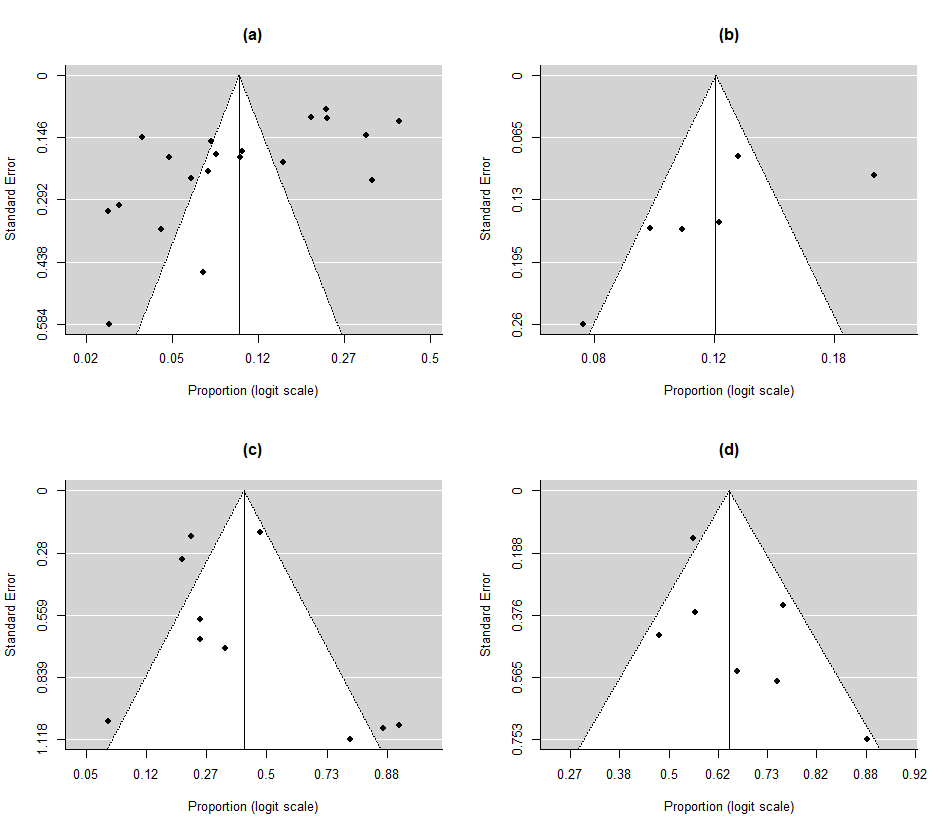


**Fig S4. Funnel plots.** Funnel plots of (a) the lesion-level prevalence of rim lesions (b) the lesion-level prevalence of chronic active lesions (c) the patient-level prevalence of rim lesions and (d) the patient-level prevalence of chronic active lesions.
