# Supplementary material for: The prevalence of paramagnetic rim lesions in multiple sclerosis: A systematic review and meta-analysis
Source: PLoS One. 2021 Sep 8;16(9):e0256845. doi: 10.1371/journal.pone.0256845 (PMC8425533; doi:10.1371/journal.pone.0256845)
Supplement: S5 File — (DOCX) [file pone.0256845.s007.docx]

**S5 File. Sensitivity analysis**


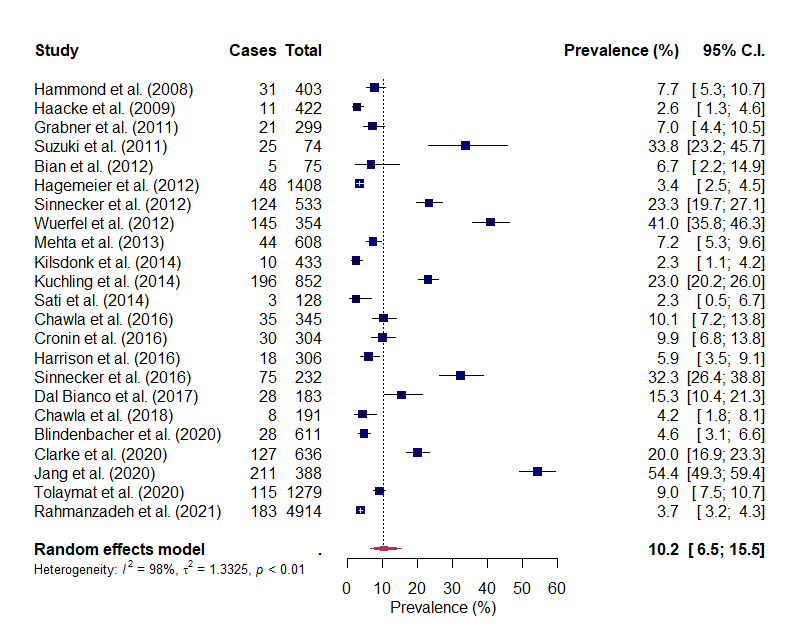


**Fig S5a.** Sensitivity analysis of the pooled lesion-level prevalence of rim lesions including three studies that post-dated our literature search.


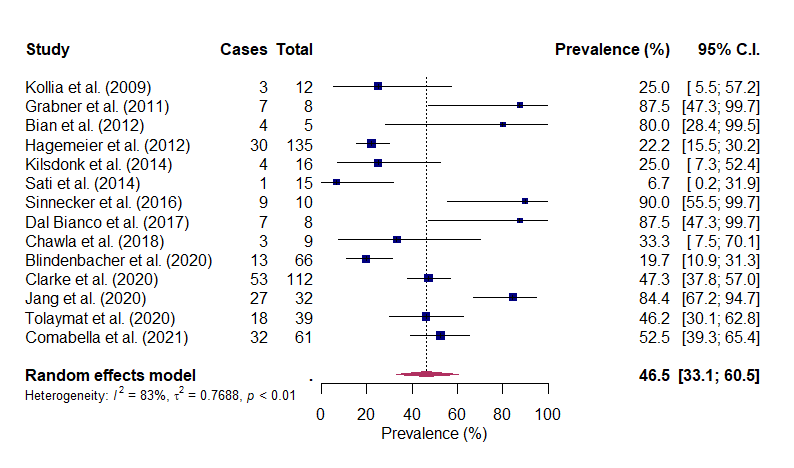


**Fig S5b.** Sensitivity analysis of the pooled patient-level prevalence of rim lesions including three studies that post-dated our literature search.


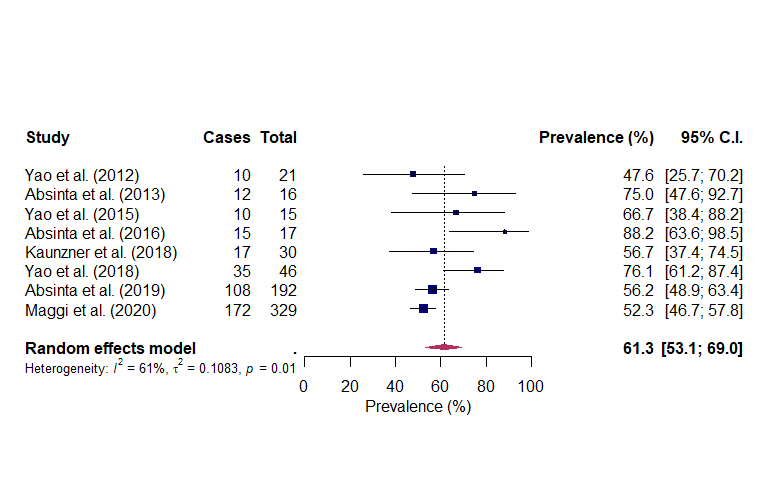


**Fig S5c.** Sensitivity analysis of the pooled patient-level prevalence of chronic active lesions including one study that post-dated our literature search.
